# Collecting Contributions for a Critical Issue: Progressing from Bench to Bedside in Bipolar Disorders

**DOI:** 10.3390/brainsci13091254

**Published:** 2023-08-28

**Authors:** Rebecca Strawbridge

**Affiliations:** Department of Psychological Medicine, Institute of Psychiatry, Psychology & Neuroscience, PO74, King’s College London, De Crespigny Park, London SE5 8AZ, UK; becci.strawbridge@kcl.ac.uk

It was a joy reading the submissions for the *Brain Sciences* Special Issue that I edited, entitled “Bipolar Disorders: Progressing from Bench to Bedside”. This was not only due to the high calibre of the research submitted by several experts in the field, or the fascinating findings and discussions, but also a number of aspects which really do help to identify a roadmap for future translational research in bipolar disorders.

As one of the most burdensome conditions worldwide, there are numerous clinical challenges that hinder people with bipolar disorder (BD) diagnoses from living a life of good quality with extended periods of full recovery [1]. Many patients spend more time in depression than other affective states, and the options for effective and safe antidepressant treatment remain far from optimal [1]. Although we have effective interventions for mania, the long-term impairment that mania can cause is of grave concern [1]. Mixed states are common, yet under-recognised and under-researched [1]. Rapid cycling is also common and under-researched [2]. Maintenance treatment to keep patients fully well on an ongoing basis is clearly of critical importance [1]. The impairments that so many patients experience, particularly to their cognitive and psychosocial functioning, are not addressed in clinical practice and subsyndromal symptoms during these periods are also of concern, as are frequent comorbidities such as substance use and anxiety disorders, as well as physical comorbidities [1].

In the 10 articles published in this Special Issue, many relevant topics and ideas for progressing the field are addressed.

Castro Menezes et al. examine factors differentiating bipolar and unipolar depression [3], which has translational potential given the well-documented, persistently high rates of mis- and delayed-diagnosis for BD, where the most frequent misdiagnosis is unipolar major depression [4]. The authors propose that examining HPA-axis biomarkers concurrently with early life stress could be useful, perhaps in progressing to a multimodal model used to help identify risk for bipolarity in people presenting with a depressive episode [3].

A second biomarker examination argues for the potential importance of astrocytes in the pathophysiology of BD. Reviewing the current evidence to date, Dai et al. identified several studies indicating a putative role of astrocytes in BD, with few overall studies and high heterogeneity between findings, indicating the importance of further large-scale investigations where key effect-modifying variables can be considered [5]. In terms of translation, this field could help to optimise established interventions with neuroprotective effects (including on astrocytes) such as lithium, and relevant therapies under investigation, e.g., pioglitazone, and novel targets.

Two further studies assessed psychological constructs known to play a role in BD. Impulsivity is a criterion for (hypo)mania, but Santana et al. systematically reviewed the extant literature on whether impulsivity is also a *trait* characteristic; of 45 total studies, their meta-analyses indicated increased self-report, as well as objective response inhibition and attentional impulsivity compared to people without a diagnosis. They highlight, however, that the field lacks longitudinal assessments, and that increased impulsivity may not be limited to BD diagnoses [6].

Lea et al.’s systematic review focused on the associations between perfectionism and affective symptoms across the affective disorder spectrum. Their meta-analysis considered only twelve studies, finding that increased depression severity is associated with greater perfectionism in multiple sub-domains, but comparisons with (hypo)manic symptoms were few, with conflicting results. There were insufficient studies to conclude whether people with unipolar vs. bipolar affective disorders differ in (trait or state) perfectionism. These were also primarily cross-sectional examinations; further elucidation of this relationship, however, could lead to future psychological therapy modifications specifically targeting perfectionism to improve outcomes [7].

A simple enhancement of symptom monitoring may also be used to optimise BD management and outcomes: routine practice still generally does not include a well-documented record of core BD symptoms over extended periods, which hinders the ascertainment of treatment response and also impacts future care for those with BD. Porter et al. validate a long-term ‘LIFE’ evaluation to assess mood symptoms over long periods in a cohort of 137 patients with BD enrolled in RCTs. Their analyses showed that a retrospective rating of mood over 6-monthly periods using the LIFE was significantly correlated with more frequent cross-sectional mood symptom ratings, suggesting that a continuous trajectory of symptoms can be recorded without ongoing frequent assessments (although this was better for depression than mania ascertainment). This could have significant near-future implications for clinical practice [8].

Arguably, we also need to better understand the drawbacks associated with even well-understood, well-evidenced current interventions. Joseph et al. conducted a historical cohort study of thyroid disorders in people on long-term lithium therapy [9]. Lithium remains the gold-standard mood stabiliser but there are several concerns related to its side effects at the therapeutic levels [10], and much of the research neglects to examine long-term therapy [11]. Of the 154 participants with BD, 10% had an existing thyroid disorder prior to lithium therapy and almost one third of the remaining sample developed a thyroid disorder during lithium treatment. The majority of thyroid disorders were treated and—notably—the rate of lithium discontinuation due to thyroid impacts was extremely low. For most patients, thyroid illness onset occurred much more than one year after lithium initiation. The rates of thyroid illness in lithium-treated patients are concerning, but as long as these are recognised and treated, may have little impact on the decision to remain on lithium. The main implications here (apparent now) are a need to monitor thyroid closely in lithium-treated patients [9].

This brings us to the systematic review in this Special Issue by Seshadri et al., examining thyroid hormone augmentation for BD [12]. The authors identified only eight interventional studies with either T3 or T4 thyroid hormones for people with BD, despite including several designs and not restricting studies according to, e.g., baseline episode or other characteristics. Some benefits were reported for people with rapid cycling and/or depression, but these appear to be mostly restricted to T4, non-RCT studies. On the other hand, thyroid hormones have been well-evidenced for unipolar depression and are one of the better-tolerated options [12].

Two other articles examine interventions with a strong evidence base for unipolar depression but less of an evidence base for BD. Firstly, Wright et al. conducted a case series of behavioural activation (BA) adapted for bipolar depression, although this was a case series with a difference, as the authors randomised patients to variable waitlist periods before/after BA [13]. The article reported high adherence (11/12 therapy completion) and acceptability (8/8 satisfied with therapy), with encouraging indications of symptom improvement (7/9 reliable improvement). Adverse events were common but judged to be unrelated to the study/therapy and the authors suggest that BA needs thorough investigation in larger, controlled clinical trials [13].

Another intervention with a large amount of supportive evidence in unipolar depression is ketamine. Youshay et al. consider the evidence base for people with bipolar depression via a scoping review [14]; overall, the 10 studies (50% RCTs) suggested that ketamine is tolerable, with a low manic switch rate, although high dropout was reported in open-label investigations. Studies were limited to IV ketamine formulation of often short durations in a small number of patients, with insufficient blinding and where many participants were undergoing continuation medications. Depression response was not reported for all studies but appeared encouraging; effects on suicidality were heterogeneous, and single studies reported potential effectiveness for fatigue and cognition. As above, the article called for substantive investigation in robust clinical trials [14].

In a final scoping review, my colleagues and I considered whether acetazolamide could have a place in the future treatment of BD [15]. We were cognizant beforehand that this evidence base is far more preliminary: [16] of any study administering acetazolamide to any person(s) with BD and reporting any clinical/acceptability/tolerability information, only five were identified, three of which were case reports. From a total of 50 treated participants, there was an estimated 44% response rate with acetazolamide (across different affective states), with a further 22% experiencing some benefits, and indications of acceptability and tolerability. Despite the clear uncertainty, given the outstanding treatment challenges in BD, it was concluded that acetazolamide should be further examined for its putative role in managing BD [15].

Commonly, these 10 articles contained increased sample sizes compared to older studies either through primary research studies [3], secondary analyses from multiple RCTs [8], the utilisation of large, well-defined medical records cohorts [9], or undertaking scoping reviews [5,14,15], systematic reviews and/or meta-analyses [6,7,12]. Where large samples were not justified, an innovative randomised case series approach was employed [13].

This Special Issue focuses on key important questions, including how to improve diagnostic specificity for BD [3,5,6], and understand its pathophysiology using biological, [5] psychological [6,7] or multimodal assessments [3]. These latter questions could help to identify novel treatment targets; other articles utilised extant pathophysiological information to examine existing interventions [12], repurposing [14] and/or modifying [13] therapies from associated disorders (largely unipolar depression), or repurposing from non-psychiatric conditions [15]. Two studies may have particular near-future clinical applications through the better recording of symptom trajectories [8] and detection of negative effects of widely used treatments [9]. The latter two also contain the dual benefits of parsimony and a focus on the long-term, which has been traditionally neglected in our field.
